# AI-enabled integrated employment ecosystem for socially vulnerable groups: a multiple case study and design-research approach

**DOI:** 10.3389/fsoc.2026.1891969

**Published:** 2026-08-25

**Authors:** Bahl-Geun Roh

**Affiliations:** Division of Business Administration, Sookmyung Women's University, Seoul, Republic of Korea

**Keywords:** AI-enabled employment, digital inclusion, hybrid organizing, multiple case study, socially vulnerable groups, sociology of work, structural exclusion

## Abstract

Population aging and digital transformation are intersecting forces that intensify the structural exclusion of older adults and persons with disabilities from contemporary labor markets. Welfare systems and scholarly literature alike remain fragmented along single-group axes, leaving this compounded inequality without an integrative response. Drawing on a multiple case study of Testworks, Kakao, and Microsoft, combined with a design-research approach, this study identifies three mechanisms through which artificial intelligence enables inclusive employment: strength matching, education-to-employment pathways, and multi-stakeholder collaboration. Cross-case analysis reveals that although all three organizations deploy AI to widen labor-market access, none integrates both vulnerable groups within a single sociotechnical system. From this shared gap, the study develops a three-stage circular ecosystem model (Education–Matching–Sustainability) anchored by a triple-layered revenue architecture. The model is presented as a conceptual and theoretical framework grounded in cross-case analysis; its practical effectiveness remains to be tested through subsequent pilot implementations and primary empirical research. The contributions are threefold. First, it specifies a sustainability architecture that transcends the mission-market dichotomy long debated in research on hybrid organizing. Second, it reframes AI as an instrument of social inclusion rather than displacement, reconnecting technology to the sociology of work and inequality. Third, it offers a transferable template for societies facing parallel demographic and digital transitions. The model thus contributes both a theoretical advance and a practical roadmap for confronting compounded forms of labor-market exclusion.

## 1 Introduction

South Korea crossed the 20% elderly threshold in 2024 and became, in formal terms, a super-aged society (Ministry of Data and Statistics, 2025). The transition took 7 years and 4 months, less than half the time Japan needed a generation earlier (OECD, 2020). It coincided with an equally rapid digital transformation that has been rewriting job structures and converting gaps in technology access into inequalities of economic participation (Ministry of Science and ICT and National Information Society Agency, 2025). Korea is not alone in this. Much of East Asia, Europe, and the developing world will face the same combined demographic and digital pressures within the next decade (World Economic Forum, 2023), and Korea provides an early and well-documented instance of how they interact.

At the center of this transformation sit two groups that bear a disproportionate share of its costs: older adults and persons with disabilities. Both groups lag on digital competencies and face labor markets that have rapidly shifted the skills in demand. The employment rate for persons with disabilities in Korea stood at 34.9% in 2024, roughly 27 points below the national average, and sheltered-workshop wages averaged KRW 500,000 per month (Ministry of Employment and Labor, 2024). Older adults have fared little better. Most remain confined to simple-labor positions under the Senior Employment Program, with monthly stipends of about KRW 270,000 (Ministry of Health and Welfare, 2025). A fragmented welfare architecture has done little to resolve what is, in effect, a compounded exclusion.

Sociologically, these patterns are not best understood as a transitional friction that labor markets will eventually absorb. Older adults and persons with disabilities encounter what Walker (2002) describes as the institutional underdevelopment of productive aging, and what Oliver (1996) frames as disability produced through social rather than individual barriers. The digital transition compounds these dynamics: Helsper (2012) shows that exclusion from digital participation translates directly into exclusion from social, economic, and civic life. The Korean case therefore offers more than an empirical setting; it represents a critical site at which the sociology of work, the sociology of disability, and the sociology of digital inequality converge in observable institutional form.

Existing welfare policies administer support for the two groups through separate institutional channels. The Senior Job Portal (MHW), the Disability Employment Portal (MOEL), and the Digital Competency Center (MSIT) each operate independently, and there is little structural basis for cross-policy synergy (Korea Social Enterprise Promotion Agency, 2023a). The academic literature mirrors this fragmentation. Research on AI and older-adult employment has accumulated on one track; research on AI and disability employment has accumulated on another. Studies that engage both populations within a single AI-mediated framework remain scarce.

Conceptually, the literature on AI and the employment of vulnerable groups has moved along two opposing tracks. The displacement view holds that automation disproportionately eliminates routine tasks and therefore threatens workers whose digital competencies are limited (Acemoglu and Restrepo, 2022). The augmentation-and-inclusion view, by contrast, emphasizes AI-assistive technologies that enhance job performance and open new employment pathways (International Labour Organization, 2023; OECD, 2020). Yet no existing model integrates AI as a bridge between the complementary capabilities of older adults and persons with disabilities.

Two research questions follow. First, what complementary capabilities and structural limitations characterize existing AI-based employment models that target socially vulnerable groups? Second, how can those complementary capabilities be combined into a single, sustainable employment ecosystem? To address these questions, this study draws on Yin’s (2014) multiple case study methodology, analyzes three organizations representing theoretically distinct approaches (Testworks, Kakao, and Microsoft), and develops a design-based integrated employment model grounded in Hybrid Organization Theory (Battilana and Lee, 2014).

The paper advances three claims. Empirically, an integrated employment framework built on the complementary capabilities of older adults and persons with disabilities is both feasible and more effective than the single-group approaches that currently dominate practice; bridging the two literatures produces insights neither could generate alone. Conceptually, AI is better read as an instrument of inclusion than as a driver of displacement or augmentation: with the right job design, curriculum, and interface, it becomes infrastructure that makes latent capabilities visible and economically deployable. Theoretically, this study sharpens Hybrid Organization Theory by specifying a triple-layered revenue structure in which public, market, and ESG-aligned logics are deliberately separated rather than fused, which directly addresses the sustainability problem that has long shadowed the literature on hybrids.

The paper proceeds as follows. Section 2 reviews the relevant literatures. Section 3 describes the research design. Section 4 presents the findings. Section 5 develops the ecosystem model and policy implications. Section 6 discusses theoretical contributions, limitations, and future directions. Section 7 concludes.

## 2 Literature review

### 2.1 Older-adult employment and the digital divide

Older-adult employment has attracted growing scholarly attention against the backdrop of global population aging, and the sociological literature on productive aging frames this attention in distinctive terms. Walker (2002) argues that productive aging is less an individual outcome than an institutional construct: whether older adults remain economically and socially active depends on how labor-market institutions, welfare regimes, and cultural scripts allocate or restrict opportunity. In a synthesis of 380 empirical studies, Ng and Feldman (2008) showed that age is positively associated with several dimensions of job performance, including interpersonal skills, organizational commitment, and reliability. Subsequent OECD (2023) data corroborate these patterns: older workers exhibit superior judgment in social interaction and crisis management, competencies that distinguish them from younger cohorts. The OECD’s (2025) Employment Outlook further documents that population aging now poses a first-order challenge to labor-market sustainability, and that AI is reshaping skill demand—raising the need for digital competencies while displacing routine cognitive and clerical tasks—a shift that bears directly on older workers with below-tertiary education. Yet these strengths must be matched by appropriate work design and lifelong learning systems—precisely the institutional supports that fragmented welfare arrangements typically fail to provide.

Theoretically, van Dijk’s (2006) digital-divide framework remains instructive. Beyond first-level divides in device access, it identifies second-level divides in digital skills and usage and third-level divides in the tangible outcomes people derive from digital engagement. In Korea, the elderly share of the population is projected to reach 47.7% by 2072 (Statistics Korea, 2023). The Senior Employment Program has expanded, yet its average monthly stipend of approximately KRW 270,000 (Ministry of Health and Welfare, 2025) is too small to support genuine economic independence. Public-interest activity programs account for roughly 74% of program types; market-oriented programs (8.3%) and job-placement programs (5.1%) together make up a marginal share (Ministry of Health and Welfare, 2025). According to the, 2024 Survey on the Digital Divide, older adults’ digital-literacy level stands at only 69.9% of the general population (Ministry of Science and ICT and National Information Society Agency, 2025).

The intersection of population aging and digital transformation generates a double-exclusion dynamic: older adults are pushed out of traditional employment sectors by technological disruption and, at the same time, locked out of emerging digital sectors by inadequate digital competencies. Age discrimination in hiring and the narrow reach of existing government retraining programs, which tend to emphasize short-term public-service activities rather than durable market-oriented employment, compound the problem (Korea Social Enterprise Promotion Agency, 2023a). The challenge, then, is to design models that close the digital skills gap while mobilizing older workers’ accumulated human capital for roles that are both meaningful and adequately compensated.

### 2.2 Employment of persons with disabilities and structural barriers

The social model of disability (Oliver, 1996) recasts disability as a product of societal barriers rather than individual deficits, redirecting analytic attention from personal impairment to the institutional, environmental, and attitudinal arrangements that produce exclusion. From this sociological standpoint, employment outcomes for persons with disabilities are best read not as expressions of individual capability but as outputs of how labor markets, employers, and assistive systems are organized. Supported-employment approaches operationalize this insight by reconfiguring workplaces and providing on-the-job support, and have been shown to outperform traditional sheltered workshops in competitive labor markets (Bond et al., 2001; Wehman et al., 2014). Strengths-based perspectives further demonstrate that persons with intellectual and developmental disabilities exhibit distinctive cognitive strengths, including pattern recognition and sustained focus, that yield superior performance in tasks requiring such qualities (Niemiec et al., 2017). These findings reinforce the sociological claim that exclusion is institutional rather than intrinsic.

In Korea, the economic-activity participation rate among registered persons with disabilities stood at 38.2% in 2024, with an employment rate of 34.9%, approximately 27 percentage points below the national average (Ministry of Employment and Labor, 2024). Average monthly wages in vocational rehabilitation facilities come to roughly KRW 500,000 (Ministry of Health and Welfare, 2023). When the figures are broken down by disability type, persons with developmental disabilities display distinctive occupational strengths, including high concentration and sustained capacity for rule-based tasks, yet systematic job-design models that build on those strengths remain underdeveloped (Korea Employment Agency for Persons with Disabilities, 2024). The digital-informatization level of persons with disabilities stands at 82.2% of the general population (Ministry of Science and ICT and National Information Society Agency, 2025). Taken together, these figures point to latent capabilities that conventional labor-market institutions have not effectively mobilized.

### 2.3 AI, employment, and digital inclusion

From a sociological perspective, the digital divide is best understood not merely as differential access to technology but as a corresponding inequality of social participation: as Helsper (2012) argues, digital exclusion translates directly into exclusion from economic, social, and civic life. Within the social-enterprise context, AI performs three core functions that work against this dynamic: customized job design anchored in individualized strength assessment, an education-employment linkage through adaptive learning systems, and data-driven quality management. Recent empirical evidence from Korean, European, and Japanese social enterprises (Shin et al., 2024; OECD, 2023) suggests that AI-enabled strength-based job design improves employment retention and productivity for vulnerable groups by approximately 20–30% compared with traditional matching approaches. Cross-national empirical work reinforces this reading while adding nuance. Analyzing 33 high-income countries, Omri et al. (2025) show that AI and digitalization significantly strengthen the link between educational attainment and employability for persons with disabilities. Abid et al. (2024), examining linear and nonlinear effects, find that AI’s employment impact on this population reverses from negative to positive beyond critical adoption thresholds—evidence that outcomes hinge on how, not whether, AI is deployed. The 2024 Digital Information Gap Survey (Ministry of Science and ICT and National Information Society Agency, 2025) reports that the comprehensive digital information level of older adults reaches only 71.8 percent of the national average. This gap is not a residual lag awaiting natural correction; it is a stabilized inequality that requires deliberate institutional intervention. Internationally, IEEE 7003–2024 has formally specified algorithmic-bias considerations for AI systems, while the EU AI Act and the United States National AI Initiative Act jointly establish the regulatory infrastructure within which inclusive AI employment can develop.

### 2.4 Hybrid Organization Theory and sustainable social enterprise

The present study draws on Hybrid Organization Theory, which conceptualizes organizations that pursue social and economic value simultaneously (Battilana and Lee, 2014). Hybrids face inherent tensions between mission logic and market logic. The conventional response is selective coupling, in which the organization adopts symbolic elements from both logics while decoupling them in practice (Pache and Santos, 2013). A more durable response is to design revenue architectures that operationalize both logics at once, combining government subsidies, market sales, and ESG-aligned partnerships (Korea Social Enterprise Promotion Agency, 2023b). Recent evidence underscores the stakes of this design choice. In a study of social-enterprise digitalization, Hu et al. (2026) find that technological change can reactivate rather than resolve hybrid tensions, reproducing the very mission-market conflicts it was expected to ease—a finding that motivates revenue architectures which separate competing logics by design rather than fusing them. Consistent with this view, Chatterjee et al. (2022) demonstrate that dynamic capability significantly predicts organizational sustainability, underscoring the value of adaptive rather than static revenue architectures. Paradox theory (Smith and Lewis, 2011) further illuminates the condition, arguing that hybrid tensions should not be resolved through trade-offs but managed through dynamic equilibration across contradictory yet interdependent demands. This lens informs the triple-layered revenue structure developed below.

### 2.5 Research gap and international perspective

The relevant research streams have expanded considerably, yet they remain largely fragmented. A recent bibliometric retrospective by Kaurav and Gupta (2022) identifies digital transformation, sustainability, and social inclusion as among the most salient emerging themes in multidisciplinary management research, while also showing that integrative frameworks bridging these themes for vulnerable populations remain underdeveloped. The present study addresses that gap by developing an integrated, AI-based employment ecosystem grounded in cross-case analysis. Internationally, a small but growing line of work has begun to examine the intersection of AI and inclusive employment. In Europe, the European Disability Strategy 2021–2030 explicitly calls for digital technologies to expand employment opportunities for persons with disabilities (European Commission, 2021), but operational models that integrate multiple vulnerable groups remain scarce. In the Asia-Pacific region, Japan’s Society 5.0 initiative (Cabinet Office of Japan, 2019) and Australia’s NDIS technology programs have produced targeted interventions for either older adults or persons with disabilities, yet none proposes an integrated model combining both populations within a single AI-enabled ecosystem. For emerging economies navigating parallel demographic and digital transitions, such a model offers both theoretical grounding and practical guidance. Table 1 summarizes the theoretical foundations that inform the analytical framework.

**Table 1:** Summary of theoretical foundations.

Theoretical stream	Key contribution	Representative scholars	Application in this study
Productive aging	Reframes older adults as active economic contributors rather than passive welfare recipients	Bass and Caro (2001) and Ng and Feldman (2008)	Grounds the Senior Teacher and mentor roles in Stages 1–2
Social model of disability	Locates disability in societal barriers rather than individual deficits	Oliver (1996) and Bond et al. (2001)	Motivates strength-based job design for persons with disabilities
Digital-divide framework	Distinguishes access, skills/usage, and outcome divides	van Dijk (2006) and Shin and Lee (2019)	Structures the Education stage curriculum
AI-employment debate	Contrasts displacement with augmentation/inclusion perspectives	Acemoglu and Restrepo (2022), International Labour Organization (2023), and OECD (2020)	Reconceptualizes AI as an instrument of inclusion
Hybrid Organization Theory and Paradox	Theorizes simultaneous pursuit of social and economic value	Battilana and Lee (2014), Pache and Santos (2013), and Smith and Lewis (2011)	Underpins the triple-layered revenue architecture

Source: Author’s compilation.

## 3 Methodology

### 3.1 Research design

This study employs a multiple case study methodology (Yin, 2014) in combination with a design-research approach. The case study component investigates how and why questions within complex real-world contexts (Eisenhardt and Graebner, 2007); the design-research component develops a prescriptive model grounded in empirical insights (van Aken, 2004). This dual methodology fits the topic because AI-based employment models for vulnerable groups are emergent phenomena that call for both analytical understanding and actionable solutions. In constructing the case protocol, the study follows Ghosh's (2025) FBR 7-String Framework for qualitative case study development, which treats theoretical framing, case selection logic, evidentiary triangulation, and transparent analytic procedure as interrelated markers of case-study rigor.

### 3.2 Case selection and justification

Three organizations were selected on three criteria: (a) a core activity involving the employment of vulnerable groups through AI; (b) applicability of the three-dimensional analytical framework; and (c) theoretical diversity across organizational form and national context. In line with Yin’s (2014) replication logic, the three cases represent theoretically distinct models: direct employment (Testworks), education-based empowerment (Kakao), and technology-platform provision (Microsoft). The selection enables theoretical replication rather than sampling-based generalization. The cases also span two institutional contexts: a rapidly aging East Asian economy undergoing aggressive digitalization (Korea) and a global technology platform operating across multiple regulatory regimes. That contextual diversity strengthens the transferability of the resulting model to other settings, including emerging economies facing parallel transitions.

The selection also reflects a design-oriented rationale specific to the model this study seeks to develop. Following Eisenhardt and Graebner’s (2007) theoretical sampling principle—cases chosen because they are particularly suitable for illuminating and extending relationships and logic among constructs—each organization represents one indispensable building block of the proposed integrated ecosystem. Testworks illustrates how strength-based job design translates into market-competitive employment for persons with disabilities, and thereby anchors the model’s Matching stage. Kakao demonstrates how learner-to-educator progression converts older adults from welfare recipients into paid contributors, and thereby anchors the Education stage. Microsoft exemplifies the multi-stakeholder infrastructure and technology-platform architecture through which such a system can scale beyond any single organization, and thereby anchors the model’s Sustainability stage. No two of these cases together would provide sufficient variation to develop the integrated model: only the three-way combination surfaces the complementary building blocks the ecosystem requires. This case-to-component correspondence is elaborated in Section 5, and its logical consistency is verified in Table 2.

**Table 2:** Model component-evidence-theory mapping (logical-consistency check).

Model component	Case evidence	Theoretical premise	Design rationale
Strength-based job design for persons with disabilities	Testworks’ developmental- and hearing-disability task allocation	Social model of disability; strength-based approach	Optimizes productivity by matching tasks to disability-type-specific capabilities
Senior Teacher and mentoring role for older adults	Kakao’s peer-to-peer senior-digital-education program	Productive aging; experiential knowledge transfer	Reframes older adults as active economic agents
Multi-stakeholder ecosystem partnerships	Microsoft’s global NGO-partnership network	Hybrid organizations rely on multi-logic coalitions	Distributes institutional and financial risk
Triple-layered revenue structure	Testworks’ B2B revenue shift; Kakao’s CSR base; Microsoft’s ESG alignment	Paradox theory (Smith and Lewis, 2011); hybrid organizing (Battilana and Lee, 2014)	Balances stability, competitiveness, and scalability
Continuous learning loop	Evolutionary trajectory of all three cases	Adaptive capability in dynamic environments	Sustains relevance under rapid technological change

Source: Author’s compilation, following van Aken’s (2004) design-research validation logic.

### 3.3 Analytical framework

A three-dimensional analytical framework guides the cross-case analysis: (1) Business Model Design, which examines revenue structures, value propositions, and stakeholder configurations; (2) AI Technology Application, which assesses the type, function, and integration mode of AI tools; and (3) Social Impact Generation, which evaluates employment outcomes, wage levels, and inclusion effects. The framework is informed by the hybrid-organizing perspective (Battilana and Lee, 2014) and is applied uniformly across all three cases to ensure analytical consistency.

### 3.4 Data sources and analysis procedure

Data were collected from multiple sources to ensure triangulation (Yin, 2014): organizational reports and publications (annual reports, sustainability reports, program documentation); government statistical data (MOEL, MHW, MSIT surveys); media coverage (press releases, news articles, industry reports); and peer-reviewed academic literature. Data collection and verification ran from January 2024 to September 2025, with the most recent authoritative figure used whenever multiple reporting periods existed. Table 3 details the data sources for each case organization.

**Table 3:** Data sources by case organization.

Case	Primary organizational sources	Government/statistical sources	Scholarly and media sources
Testworks	Asan Nanum Foundation report (2025); company disclosures (2023–2024)	MOEL disability employment statistics (2024); KEAD reports (2024)	Shin et al. (2024); Stanford Social Innovation Review
Kakao	MHW–Kakao cooperative agreement documentation (2024)	MHW Senior Employment Program guidelines (2025); MSIT-NIA digital-divide survey (2025)	Peer-reviewed articles on Korean senior education; reputable press coverage
Microsoft	AI for accessibility program reports; Microsoft sustainability disclosures	OECD digital-inclusion reports (2020); UN CRPD implementation briefs	Academic literature on assistive AI; global technology press

Source: Author’s compilation.

Coding proceeded through three iterations structured by the three-dimensional analytical framework (Miles et al., 2020). In the first, open-coding iteration, source materials for each case were segmented and labeled against the three primary dimensions—Business Model Design, AI Technology Application, and Social Impact Generation—with each data segment tagged to its originating source to preserve the audit trail. In the second, axial-coding iteration, first-order codes were grouped into higher-order categories: within the AI-technology dimension, for example, the codes “competency diagnostics,” “adaptive learning,” and “accessibility API” were grouped under the category of strength-identification mechanisms. In the third, pattern-coding iteration, categories recurring across all three cases were elevated to cross-case themes—the three commonalities and two differences reported in Section 4.2. Discrepant evidence was retained rather than discarded: where a source diverged from the emerging pattern, the divergence was logged and resolved against the most recent primary-organizational disclosure, following the corroboration rule specified in Section 3.5. Because a single investigator conducted the coding, each coding decision was documented with its source anchor so that the reasoning could be independently audited, a safeguard discussed further in Section 3.5. The analysis proceeded in two phases. In the first, within-case phase, each organization was analyzed independently along the three framework dimensions, producing a structured within-case narrative that documented business-model configuration, AI-application mode, and social-impact outcomes together with their evidentiary sources; this phase corresponds to the three within-case accounts in Sections 4.1.1 through 4.1.3. In the second, cross-case phase, the three within-case narratives were compared dimension by dimension to surface convergent and divergent patterns, following a matrix logic in which each case occupied a row and each analytical dimension a column, so that commonalities and differences became visible as column-wise regularities and contrasts; this phase produced the synthesis in Section 4.2, from which the integrated employment model was derived. To strengthen analytical rigor, the study applied a pattern-matching logic (Yin, 2014): observed patterns, including the use of AI for strength identification, the coupling of education and employment, and the reliance on multi-stakeholder networks, were compared with theoretically predicted patterns derived from Hybrid Organization Theory and the strength-based employment literature. Rival explanations, including pure CSR-driven philanthropy and conventional subsidy-dependent social enterprise, were considered and assessed against the evidence. Convergence between observed and predicted patterns across the three cases strengthens the analytical generalization of the findings.

### 3.5 Trustworthiness and quality safeguards

Because the study relies exclusively on publicly available secondary data, several safeguards were built in to address the four standard trustworthiness criteria for qualitative inquiry (Lincoln and Guba, 1985). First, credibility was strengthened through source triangulation across four evidence types: organizational reports, government statistical releases, peer-reviewed academic literature, and reputable media coverage. The approach cross-validated claims across channels with differing incentive structures. All quantitative indicators (employment figures, wage levels, program participation rates, and investment amounts) were corroborated against at least two independent sources wherever feasible, and discrepancies were resolved by giving priority to the most recent primary-organizational disclosure. Second, transferability was enhanced through thick description of each case context. Third, dependability was supported by an audit trail linking each coding decision to a specific source within the three-dimensional protocol. In this study, that trail linked each of the three coding iterations—open, axial, and pattern coding, as described in Section 3.4—to the specific sources on which each decision rested. Fourth, to address confirmability, interpretive judgments were anchored to direct organizational disclosures, and rival explanations were actively tested against the evidence. Access dates were recorded for all time-sensitive statistics to ensure data transparency. These procedures do not eliminate the limitations inherent in secondary-data analysis, but they substantially reduce the risk of unsupported interpretation.

## 4 Findings

Before presenting the findings, a note on their epistemic status is in order, since the analysis moves across four distinct registers that should not be conflated. Empirical findings are statements about what the cases actually show—employment figures, wage levels, revenue structures, and documented operational practices drawn from the sources in Table 3. Conceptual assumptions are the analytic premises the study brings to those findings, most notably the notion of complementary convergence introduced in Section 5. Theoretical interpretations are claims about what the findings mean when read through the frameworks of Section 2—for instance, the reading of AI as inclusion-enabling infrastructure rather than labor substitute. Normative recommendations, developed in Section 5.5, are claims about what actors ought to do. This section confines itself to the first and, where explicitly marked, the third register; conceptual and normative content is reserved for Section 5. Throughout, interpretive claims are signaled by explicit markers to keep them distinct from the observations they rest on.

### 4.1 Within-case analysis

#### 4.1.1 Testworks: AI data production centered on persons with disabilities

##### 4.1.1.1 Business model insight

Testworks was founded in 2015 by a former Microsoft and Samsung engineer and has built its core business around AI training-data collection, processing, and quality assurance, delivered under B2B contracts with private-sector firms. Its revenue is composite: corporate contract fees sit alongside government employment subsidies. The client base now exceeds 50 firms spanning multinationals and domestic conglomerates. This commercial footprint demonstrates that disability employment, when properly designed, can compete in the AI data value chain on market terms (Asan Nanum Foundation, 2025).

##### 4.1.1.2 AI technology application insight

Testworks distinguishes itself not through the AI it uses but through how it assigns work. Workers with developmental disabilities are directed to image classification, labeling, and pattern-tagging—tasks matched to their characteristic concentration and tolerance for repetition—through a systematic job design protocol. Workers with hearing disabilities are routed to video-content quality assurance, which benefits from their heightened visual attention (Shin et al., 2024). A proprietary AI diagnostic tool tracks processing speed, accuracy, and task preference at the individual level, so that task allocation adjusts as workers develop and as client requirements shift.

##### 4.1.1.3 Social impact insight

By 2023, Testworks employed 28 persons with disabilities (17 with developmental disabilities and 11 with hearing impairments) at an average monthly wage of roughly KRW 1,400,000, about 2.8 times the sheltered-workshop level (Shin et al., 2024). Investment had exceeded USD 8 million, and revenue was on track for USD 9 million by year-end. Three limitations stand out: no older-adult participation, a narrow range of covered disability types, and operations concentrated in the Seoul metropolitan area.

#### 4.1.2 Kakao: older-adult digital educator cultivation model

##### 4.1.2.1 Business model insight

The Senior Digital School is Kakao’s attempt to fuse its technological capabilities with social infrastructure. It runs as a public-private partnership, jointly funded by CSR allocations and MHW cooperative projects (Ministry of Health and Welfare, 2024), and is built around a single value proposition: older adults should not merely consume new technology; they should teach it.

##### 4.1.2.2 AI technology application insight

Two elements make up the program. An adaptive learning system diagnoses each learner’s digital-competency level and tailors content accordingly. A peer-to-peer model then draws on the empathic communication that same-generation educators bring, which lowers the anxiety that often accompanies digital learning in older adults and improves retention relative to conventional instructor-learner pairings.

##### 4.1.2.3 Social impact insight

Cumulative enrollment has reached roughly 10,000, of whom 1,200 (12%) have moved into the Senior Teacher role. Average digital competency has improved by 35%. The model’s weaknesses are equally visible: it excludes persons with disabilities, and its heavy reliance on CSR budgets leaves both scalability and institutional entrenchment in doubt.

#### 4.1.3 Microsoft: global technology support model

##### 4.1.3.1 Business model insight

Microsoft operates at a fundamentally different scale. Through its AI for Accessibility program, the firm has committed USD 25 million over 5 years, channelled through a worldwide nonprofit partnership network. The program is structured as an open platform rather than as a direct-employment vehicle, which reflects a technology-provision logic quite different from the logics at work in the two Korean cases.

##### 4.1.3.2 AI technology application insight

The tool portfolio is broad: Seeing AI for visual disabilities, real-time captioning for hearing disabilities, eye-tracking interfaces for physical disabilities, and Immersive Reader for learning disabilities. All are exposed as APIs and embedded in third-party employment and education applications worldwide, which is the mechanism through which the firm’s reach is multiplied.

##### 4.1.3.3 Social impact insight

The program has secured more than 500 NGO partnerships globally and is aligned with CRPD implementation and the UN Sustainable Development Goals. What it does not do is link people directly to employment, nor are its solutions optimized for the Korean institutional context. Both facts limit how readily the model can be transplanted into domestic inclusive-employment systems.

### 4.2 Cross-case synthesis

The cross-case synthesis identified three commonalities and two key differences.

#### 4.2.1 Commonality 1: AI as an enabler of strength-based inclusion

All three organizations use AI as a means of identifying, matching, and amplifying the strengths of vulnerable populations, whether through competency diagnostic tools (Testworks), adaptive learning systems (Kakao), or accessibility APIs (Microsoft). Across the three cases, AI functions not as a substitute for human labor but as an infrastructure that makes latent capabilities visible and deployable in economic terms.

#### 4.2.2 Commonality 2: the education–employment nexus

In every case, sustainable employment presupposes prior investment in competency development. Testworks runs an on-the-job training program; Kakao builds an education-employment cycle; Microsoft delivers an implicit training infrastructure through its partner organizations. The coupling of learning and work, in other words, appears to be a structural feature of AI-enabled inclusive employment rather than an optional add-on.

#### 4.2.3 Commonality 3: multi-stakeholder collaboration

Each model depends on a network of stakeholders, including government agencies, private-sector clients, and NGO partners. The pattern suggests that vulnerable-group employment in the AI era is inherently a collaborative, ecosystem-level endeavor rather than a single-firm initiative.

#### 4.2.4 Difference 1: direct employment vs platform provision

Testworks and Kakao directly employ or train vulnerable populations, whereas Microsoft provides enabling technology without direct employment linkage. The divergence reflects distinct organizational logics: operational inclusion (Testworks/Kakao) versus infrastructural inclusion (Microsoft).

#### 4.2.5 Difference 2: single-group focus vs integrated approach

None of the three cases integrates older adults and persons with disabilities within a single employment ecosystem. This convergent limitation strongly motivates the integrated model proposed below.

Consistent with Yin’s (2014) pattern-matching logic, two rival explanations for the observed patterns were explicitly tested and rejected. The first holds that the observed employment outcomes can be adequately explained by conventional CSR-driven philanthropy. This explanation fails on three grounds: (a) Testworks derives the majority of its revenue from B2B market contracts rather than donations, which indicates a market-competitive rather than philanthropic mechanism; (b) Kakao’s Senior Digital School institutionalizes paid educator roles with measurable competency outcomes, going beyond what typical CSR programs deliver; and (c) Microsoft’s AI for Accessibility platform, though CSR-funded at inception, operates through open-source APIs that create structural rather than transactional value. The second rival holds that the cases represent conventional subsidy-dependent social enterprises whose outcomes are driven primarily by public funding. This interpretation is similarly insufficient: Testworks’ revenue mix shows a progressive shift from reliance on subsidies toward market-based income, and Microsoft operates without Korean public subsidies at all. Rejecting these rivals reinforces the theoretical premise that AI-enabled, strength-based employment constitutes a distinct mechanism, one that cannot be reduced to either philanthropy or subsidy dependence, and it strengthens the causal case for the integrated-ecosystem model developed in Section 5 (see Table 4).

**Table 4:** Comparative analysis of case organizations.

Dimension	Testworks	Kakao senior digital school	Microsoft AI for accessibility
Target group	Persons with disabilities (developmental, hearing)	Older adults (55+)	Persons with disabilities (cross-type)
Business model	B2B market contracts + government subsidies	CSR + MHW public-private partnership	Global platform with NGO partnerships
Primary AI role	Competency diagnostics and strength-based task allocation	Adaptive learning and peer-to-peer mentoring support	Accessibility APIs embedded in third-party applications
Employment outcome	28 employees; approx. KRW 1,400,000 monthly wage	Approx. 1,200 Senior Teachers; KRW 300,000–500,000 monthly stipend	500+ NGO partnerships globally; no direct employment
Key limitation	Narrow disability-type coverage; regional concentration	Exclusion of persons with disabilities; CSR dependence	No employment-linkage mechanism; limited Korean-context fit

Source: Author’s compilation based on organizational reports, government data, and scholarly sources. The Kakao compensation figure (KRW 300,000–500,000 per month) represents a monthly stipend for Senior Teacher participants within the MHW Senior Employment Program rather than a conventional market wage; direct comparison with Testworks’ full-time wage should be interpreted with this structural distinction in mind (Ministry of Health and Welfare, 2025).

## 5 Model development: an AI-enabled integrated employment ecosystem

### 5.1 Model concept: a three-stage circular ecosystem

The cross-case analysis motivates an AI-based integrated employment model organized around three sequential stages. Stage 1 (Education) trains older adults in digital competencies and prepares them for roles as Senior Teachers. Stage 2 (Matching) develops AI-based occupational roles for persons with disabilities, with older adults now serving as mentors. Stage 3 (Sustainability) underwrites the whole through a triple-layered revenue structure, and experienced persons with disabilities begin to train incoming cohorts, closing the loop. Table 5 lays out each stage in detail, specifying objectives, target groups, principal activities, expected outputs, and theoretical anchors.

**Table 5:** Structural components of the three-stage circular ecosystem model.

Stage	Target group	Principal activities	Expected outputs	Theoretical foundation
Stage 1: education	Older adults (55+)	Digital-competency training; generative-AI practical training; mentoring-skills development	Senior Teachers; data labelers; quality managers	Productive aging (Bass and Caro, 2001); digital-divide framework (van Dijk, 2006)
Stage 2: matching	Persons with disabilities	Strength-based job design; AI-assisted task allocation; integrated mentoring by older adults	Employed workers with disability-type-optimized roles	Social model of disability (Oliver, 1996); strength-based approach (Niemiec et al., 2017)
Stage 3: sustainability	Ecosystem-wide	Triple-layered revenue management; peer-to-peer trainer cultivation; continuous adaptation	Self-sustaining inclusive ecosystem	Hybrid Organization Theory (Battilana and Lee, 2014); paradox theory (Smith and Lewis, 2011)

Source: Author’s compilation, integrating cross-case findings with the theoretical foundations in Table 1.

The model integrates Testworks’ task-based job design, Kakao’s learner-to-educator progression, and Microsoft’s partnership architecture, directly addressing the limitation each case shares: a focus on only one vulnerable group. The triple-layered revenue structure departs from single-source funding models by combining public support, market revenue, and ESG investment.

Before presenting the model, its epistemic status warrants explicit clarification. The three-stage ecosystem developed here is a conceptual and theoretical framework—a design-oriented synthesis derived from cross-case analysis of three operating organizations and grounded in the literatures reviewed in Section 2. It is not, and does not claim to be, an empirically validated intervention. Its components are theorized rather than tested; its projected outcomes are inferred rather than measured. In line with van Aken’s (2004) design-research paradigm, the framework’s function at this stage is to specify a coherent and testable architecture that subsequent empirical work can validate, refine, or refute. The pilot implementations and longitudinal studies through which such validation should proceed are set out in Section 6.4. The policy recommendations that follow in Section 5.5 then constitute the normative register: they specify not what the cases show or what the model means, but what public and corporate actors ought to do to realize it.

The theoretical rationale for integration rests on the concept of complementary convergence. Older adults bring experiential knowledge, interpersonal skills, patience, and mentoring capacity, attributes that have been consistently validated in the productive-aging literature (Bass and Caro, 2001; Ng and Feldman, 2008). Persons with disabilities, and particularly those with developmental disabilities, bring high concentration, sustained focus on rule-based tasks, and digital task-execution capabilities that have been shown empirically to be competitive with, and in several AI data-processing domains superior to, those of non-disabled workers (Shin et al., 2024). When these complementary capabilities are combined within a structured mentoring and collaborative work system, the resulting synergy exceeds what either group could achieve alone. This complementary convergence also addresses a persistent criticism of social-enterprise models: the tendency to treat beneficiary groups as passive recipients rather than active contributors to value creation.

Following van Aken (2004), each model component is mapped onto the case evidence that motivates it and the theoretical premise it operationalizes. Table 2 presents this mapping.

### 5.2 Stage 1: education program design

The older–adult education program targets individuals aged 55 and above and is delivered over 8 weeks (three sessions per week, 48 h in total). The curriculum covers foundational digital skills, practical training in generative AI, data-work fundamentals, and mentoring skills. On completion, participants can move into Senior Teacher, data-labeler, or quality-manager roles, which creates a clearly defined pathway from learner to paid contributor.

Education for persons with disabilities runs over 9 weeks (five sessions per week, 90 h in total) with customized curricula that accommodate different types of disability. Assistive technologies such as screen readers and voice recognition are integrated throughout. This stage represents a fundamental shift from welfare recipient to economic agent, a shift consistent with the strength-based approach documented in the literature (Niemiec et al., 2017) and illustrated in the Testworks case.

### 5.3 Stage 2: job matching program design

Four primary occupational roles are defined for older adults: digital-education instructor, data-quality manager, customer-support consultant, and content moderator. For persons with disabilities, jobs are structured to maximize disability-type-specific strengths: image classification for developmental disabilities, video-quality assurance for hearing disabilities, speech-to-text transcription for physical disabilities, and text proofreading for visual disabilities.

The core of this stage is an integrated collaborative work structure in which older adults serve as mentors overseeing data-quality assurance, while persons with disabilities carry out the substantive data processing and content production. The structure operationalizes the principle of complementary convergence by translating the abstract notion of capability complementarity into specific role assignments and workflow linkages.

### 5.4 Stage 3: revenue structure design

The triple-layered hybrid revenue structure comprises government subsidies (initial stabilization, Years 1–2), corporate B2B sales revenue (target: 50% of total revenue by Year 3), and ESG partnerships (social-impact investment, convertible into performance-based returns). Seen through the lens of Hybrid Organization Theory (Battilana and Lee, 2014; Pache and Santos, 2013) and paradox theory (Smith and Lewis, 2011), the structure integrates stability, market competitiveness, and scalability rather than trading them off against one another. Each revenue stream is explicitly aligned with a distinct institutional logic, namely public mission, market efficiency, and stakeholder-ESG governance, and the structural separation prevents any single logic from crowding out the others.

### 5.5 Policy recommendations

#### 5.5.1 Government-level recommendations

Three core initiatives are proposed: an integrated digital-employment platform consolidating MHW, MOEL, and MSIT systems; Amendment of the Social Enterprise Promotion Act to create an Older-Adult-Disability Integrated Social Enterprise category; and a Digital Inclusion Job Fund of KRW 30 billion over 3 years. The initiatives target the institutional fragmentation identified in Section 1 and provide concrete legislative and fiscal instruments through which the integrated ecosystem can be operationalized. Table 6 summarizes the recommendations.

**Table 6:** Government-level policy recommendations.

Recommendation	Key elements	Responsible authority	Expected outcome
Integrated Digital Employment Platform	Consolidation of MHW, MOEL, and MSIT systems into a single portal for older adults and persons with disabilities	MHW, MOEL, MSIT (joint governance)	Reduced institutional fragmentation; unified user experience
Amendment of the Social Enterprise Promotion Act	Creation of an Older-Adult-Disability Integrated Social Enterprise category with dedicated certification pathways	MOEL and KSEPA	Legal recognition of integrated models; dedicated funding eligibility
Digital Inclusion Job Fund	KRW 30 billion over 3 years, structured in capability-building, employment-matching, and scale-up tranches	MSIT with MOEL/MHW co-financing	Scalable public financing aligned with the three-stage ecosystem

Source: Author’s compilation.

#### 5.5.2 Corporate-level recommendations

Four incentive mechanisms are proposed: expanded tax credits (from 10 to 15%), public-procurement bonuses (+5 points in KONEPS), ESG certification benefits (+3 points in KCGS evaluation; Korea Corporate Governance Service, 2024), and government-supported brand-value enhancement. The mechanisms operationalize a straightforward business case: investing in vulnerable-group employment generates social value and, concurrently, strengthens corporate competitiveness. Table 7 details the mechanisms.

**Table 7:** Corporate participation incentive mechanisms.

Mechanism	Key elements	Implementing agency	Strategic rationale
Expanded tax credits	Corporate tax credit raised from 10 to 15% for qualifying integrated-employment expenditure	Ministry of Economy and Finance; National Tax Service	Lowers effective cost of inclusive employment
Public-procurement bonus	+5 points in the Korea ON-line E-Procurement System (KONEPS) evaluation for participating firms	Public Procurement Service	Links inclusive employment to competitive advantage in public markets
ESG certification benefit	+3 points in Korea Corporate Governance Service (KCGS) ESG evaluation	KCGS	Translates inclusive employment into capital-market signals
Brand-value enhancement	Government-supported co-branding and certification marks for integrated-employment enterprises	KSEPA with MOEL	Strengthens reputational returns for participating firms

Source: Author’s compilation.

## 6 Discussion

### 6.1 Theoretical implications

This study contributes to three theoretical conversations. The first is the long-running exchange on social enterprise and vulnerable-group employment, where research on older adults (Bass and Caro, 2001; Shin and Lee, 2019) and research on persons with disabilities (Bond et al., 2001; Oliver, 1996) have developed side by side rather than in dialogue. Complementary convergence, the idea that older adults’ experiential knowledge and mentoring capacity pair productively with the digital task-execution strengths of persons with disabilities, moves beyond the limits of single-group approaches and suggests a different way of thinking about what inclusive employment can look like.

The second conversation is about AI itself. The discourse has tended to polarize around displacement and augmentation (Acemoglu and Restrepo, 2022; International Labour Organization, 2023). The evidence suggests a third possibility: AI as a systemic enabler of inclusive employment, a role that neither the displacement nor the augmentation account captures adequately. The analytical framework used throughout, covering business model, AI application, and social impact, offers future researchers a tractable lens for studying AI’s role in vulnerable-group employment in other contexts.

The third conversation concerns hybrid organizations. The triple-layered revenue structure proposed here extends Hybrid Organization Theory (Battilana and Lee, 2014) by giving social enterprises a concrete mechanism for balancing mission and market logics, and thereby addresses the sustainability problem that has long shadowed the literature (Pache and Santos, 2013). Separating stability (government subsidies), competitiveness (market sales), and scalability (ESG partnerships) yields a more textured account than the public-private dichotomy typically used in social-enterprise research. Read through paradox theory (Smith and Lewis, 2011), the three-logic architecture operationalizes the dynamic equilibration that hybrids need across contradictory yet interdependent institutional demands, and it extends the theoretical vocabulary available for analyzing sustainable hybridity.

### 6.2 Practical implications

For policymakers and social-enterprise practitioners, the three-stage ecosystem offers a workable blueprint rather than an aspirational sketch. Its education programs come with specific curricula, defined durations, and measurable outcomes, which makes direct adoption feasible. The accompanying policy proposals, including the integrated digital-employment platform and the Digital Inclusion Job Fund, translate into implementable measures with identified authorities and tractable expected outcomes.

For firms, the incentive mechanisms laid out above make a straightforward business case: investing in vulnerable-group employment produces social value and, concurrently, strengthens competitive position through better ESG performance. The model was built in and for Korea, but its underlying mechanisms, strength-based job design, adaptive learning, and hybrid revenue structures, travel reasonably well. The European Disability Strategy 2021–2030, Japan’s Society 5.0 initiative, and Australia’s NDIS technology programs each offer institutional scaffolding into which an integrated ecosystem could be fitted (Cabinet Office of Japan, 2019; European Commission, 2021). Transferability will not be automatic, however. Differences in welfare systems, labor-market institutions, cultural norms around aging and disability, and the regulatory categories governing social enterprises will each require substantial recalibration of funding mechanisms, curricula, and stakeholder configurations.

The case for transferability is particularly strong in emerging economies. India, Vietnam, and Indonesia are all projected to face both population aging and a widening digital divide over the next decade (World Economic Forum, 2023). The ecosystem proposed here supplies a design template adaptable with two main adjustments: substituting domestic public-sector counterparts for the roles MHW, MOEL, and MSIT play in Korea, and aligning the triple-layered revenue structure with the ESG and social-impact-investment frameworks that prevail locally. Whether the transferable elements are structural principles or context-specific design parameters—a question systematized in Table 8—is ultimately one for cross-national comparative research.

**Table 8:** Contextual adaptation requirements for cross-national transfer.

Context	Institutional anchor	Required adaptations	Principal transferability limits
European Union	EU AI Act (2024/1689); European Disability Strategy 2021–2030; European Pillar of Social Rights	Alignment with high-risk AI provisions; integration with member-state disability employment quotas; ESG structure adapted to CSRD reporting standards	Fragmented labor-market institutions across member states; heterogeneous social-enterprise legal categories
Japan	Society 5.0 initiative; Basic Act for Persons with Disabilities; Silver Human Resource Centers	Interface with existing senior-employment infrastructure; recalibration of mentor-mentee ratios for higher elderly share; adaptation to keiretsu-style partnership norms	Very high elderly dependency ratio may strain the public-financing tranche; comparatively lower digital-inclusion pressure on younger cohorts
Australia	National Disability Insurance Scheme (NDIS); Fair Work Act; Modern Slavery Act	Integration with NDIS participant plans; alignment with state-level disability employment strategies; ESG structure aligned with mandatory modern-slavery reporting	Geographic dispersion complicates ecosystem density; smaller domestic market for B2B AI-data services
Emerging economies (India, Vietnam, Indonesia)	Rights of Persons with Disabilities Act (India, 2016); social-enterprise pilots under national digital-inclusion strategies	Substitution of domestic public-sector authorities for Korean MHW-MOEL-MSIT roles; alignment with locally prevailing ESG frameworks; adjustment for larger informal labor sector	Weaker regulatory infrastructure for AI governance; capital-market thinness limits the ESG-partnership tranche; higher demographic-transition volatility

Source: Author’s compilation, drawing on institutional documentation from each context.

Table 8 systematizes the contextual adaptations required for transfer across four representative settings, and identifies the principal transferability limits in each.

### 6.3 Ethical considerations

Using AI in relation to vulnerable populations raises ethical questions that demand explicit engagement with contemporary algorithmic-accountability frameworks. Transparency, fairness, and accessibility in AI-based competency-assessment tools are not optional; without them, the risk of algorithmic discrimination is real and well documented (Eubanks, 2018). Adaptive learning systems must be designed with user autonomy and dignity in mind, and must resist the temptation to collapse individuals into labels defined by disability type or age cohort. Data-privacy safeguards, finally, are essential for the sensitive personal information that AI-based diagnostic and matching systems inevitably process.

Six ethical dimensions warrant explicit treatment in an AI-enabled inclusive-employment system. Algorithmic bias arises when training data or model architectures encode historical patterns of exclusion, so that AI-based competency assessments may systematically underestimate the capabilities of workers whose profiles are underrepresented in the reference distribution. Discrimination extends this concern to downstream outcomes, where seemingly neutral matching decisions can reproduce structural inequalities across disability type, age cohort, or region. Data privacy is heightened for socially vulnerable populations, whose disability status, health information, and personal identifiers require protection under stricter than baseline standards. Transparency requires that decision logics—including how strengths are inferred and how tasks are allocated—be legible to affected workers and their advocates, not only to system designers. Explainability translates transparency into operational form: workers should be able to obtain a human-intelligible account of why a specific role, task, or training pathway has been recommended for them, and should have institutionally supported channels through which to contest that account. Accountability closes the loop by assigning identifiable persons, organizations, or bodies the duty to act when transparency reveals bias, when explainability reveals ill-founded reasoning, or when monitoring reveals disparate impact. These six dimensions together define the ethical envelope within which the proposed ecosystem must operate.

The ecosystem proposed here uses AI in employment decisions affecting socially vulnerable groups, which places it squarely within what the European Union AI Act (Regulation (EU) 2024/1689) classifies as a high-risk use case. High-risk status triggers requirements for risk management, data governance, transparency, human oversight, and conformity assessment. Implementation should therefore be aligned with the EU AI Act’s high-risk provisions for employment-related AI systems, with the IEEE 7000-series standards on ethically aligned design (IEEE 7010 for well-being metrics and IEEE 7003 for algorithmic bias; IEEE, 2020, 2024), and with instruments such as the UN Convention on the Rights of Persons with Disabilities and the OECD AI Principles. At the operational level, four safeguards matter most: third-party algorithmic audits of competency-diagnostic tools, participatory co-design with older adults and persons with disabilities, opt-out mechanisms that preserve access to non-AI-mediated pathways, and ex-post monitoring of disparate-impact indicators across disability types, age cohorts, and regions.

### 6.4 Limitations and future research

The study’s contributions come with limitations that also mark directions for future work. The most consequential is that the model has not yet been validated through pilot implementation: as a design-research study based on secondary-data analysis, it rests on what can be inferred from existing cases rather than on what is observed when the ecosystem is actually built and run.

A structured validation agenda follows from this acknowledgment. First, a small-scale pilot implementation—involving one or two operating social enterprises adopting the three-stage architecture—would generate the first-order evidence on operational feasibility, participant uptake, and revenue-mix stability. Wehman et al.’s (2014) case–control design offers a workable template for such a pilot, with treatment and comparison cohorts tracked over 12–18 months. Second, primary empirical studies—including interviews with programme participants, mentors, employer partners, and platform architects, complemented by administrative-record analysis of employment retention, wage progression, and skill acquisition—would document mechanisms that secondary sources cannot access. Third, a longitudinal design tracking cohorts across 3–5 years would test the sustainability claim at the core of the model: whether the triple-layered revenue architecture remains stable as the ratio of public, market, and ESG-aligned sources shifts over time. Cross-national comparative work—replicating the pilot in institutional settings with different welfare regimes, disability policies, and social enterprise legislation—would then test the transferability claims advanced in Section 6.2. Together, these four research streams would move the framework from a conceptual synthesis toward an empirically calibrated design tool.

Primary data collection, such as interviews with program participants or organizational stakeholders, would add contextual depth that secondary sources cannot supply. A natural next step is longitudinal pilot implementation with mixed-methods evaluation, combining quantitative performance metrics (employment rates, wages, retention) with qualitative assessments (participant satisfaction, dignity preservation, and community integration).

Three further limitations warrant note. The three case organizations represent specific organizational types and national contexts, and the findings’ generalizability to other institutional and cultural settings will require comparative international research, especially in emerging economies where demographic and digital transitions rest on different institutional foundations. The revenue projections, though grounded in existing case data, remain projections; their feasibility under varying economic conditions is ultimately an empirical question. And the analysis does not engage intra-group heterogeneity in depth. The needs of persons with mild and severe disabilities differ, as does digital readiness across age cohorts among older adults. Differentiated sub-models that take such heterogeneity seriously are an important next step.

Finally, the analysis was conducted by a single investigator. Following Lincoln and Guba (1985) and Yin (2014), four procedural safeguards addressed this limitation: interpretive judgments were anchored to direct organizational disclosures; claims required corroboration across at least two independent source types; a structured case protocol produced a traceable audit trail; and rival explanations were actively tested. Independent multi-coder analysis would further strengthen confirmability in future work.

## 7 Conclusion

This paper has developed an AI-enabled integrated employment model that treats older adults and persons with disabilities as a single population to be served rather than two separate caseloads to be processed. It departs from the fragmented, single-group approaches that currently dominate both policy and scholarship, showing how AI can serve as an enabler of inclusion rather than a driver of displacement. The three-stage circular ecosystem (Education, Matching, Sustainability) draws on the complementary capabilities each group brings, and its long-term financial viability rests on a triple-layered hybrid revenue structure that separates public, market, and ESG-aligned logics rather than forcing them to collapse into one.

The paper contributes on three fronts. It builds an integrated employment framework that brings together two research streams which have, so far, been kept apart. It reframes AI as an instrument of inclusion, making strength-based job design and adaptive learning do theoretical work that displacement and augmentation accounts cannot. And it sharpens Hybrid Organization Theory by specifying a sustainable financial architecture for social enterprises operating at the intersection of demographic change and digital transformation. Validation through longitudinal pilot implementation, with mixed-methods evaluation across diverse institutional and cultural contexts, is the obvious next move, and should include the emerging economies now facing parallel demographic and digital transitions.

Super-aging and digital transformation are remaking labor markets on every continent. The resulting pressure on inclusive employment is not a future concern; it is a present one, and it falls hardest on populations that fragmented welfare systems have historically failed. The framework developed here is grounded in theory and designed for implementation, offered as a foundation for further inquiry and practice. If AI can be deployed as an instrument of inclusion rather than displacement, and if the complementary capabilities of older adults and persons with disabilities can be combined productively within a single ecosystem, then the future of work need not be one in which technological progress and social exclusion march together.

## Data Availability

The original contributions presented in the study are included in the article/supplementary material, further inquiries can be directed to the corresponding author.
